# Inspector-General Sir James Ranald Martin, C.B., F.R.S.

**Published:** 1899-03

**Authors:** 


**Inspector-General Sir James Ranald Martin, C.B., F.R.S.** By Surgeon-General Sir JOSEPH FAYRER, Bart., M.D., F.R.S. Pp. xvi., 203. London: A. D. Innes & Co. 1897.

This memoir of Sir Ranald Martin, written by a man so well known and appreciated as Sir Joseph Fayrer, is a guarantee for a truthful account of the life of one who was distinguished not only in the position he held in India as a Medical Officer of the Indian Army, but also for his long and honourable career in civil practice both in India and in London, for the admirable work he did as Physician to the Medical Board at the India Office, and also for the excellent literary works on tropical diseases and climates which he has left behind him.

Sir Ranald Martin, like many other distinguished public servants, hailed from the west coast of Scotland, from the Island of Skye, which is as celebrated for its loyal and patriotic sons as for its gallant and brave though humble breed of wiry terriers.

He came of an ancient and honourable lineage, and, as Sir Joseph Fayrer observes, he not only well maintained the tradition of the stock from which he sprang, but he transmitted his inherited good qualities to a numerous family of sons, some of whom served with distinction in the army and in the Civil Service of India.

Ranald Martin was originally intended for the combative ranks of the army. After he had been educated at the Inverness Royal Academy, a commission was ready for his acceptance in the 42nd Highlanders, the renowned Black Watch. But another career seemed to his father to offer him better prospects. The parental resources were at that time but slender, and the prospect of having to purchase all his steps seemed to his father more than those resources would bear. Young Ranald therefore acquiesced in his father's advice, and although the disappointment was bitter he chose medicine as his future career. He began to study first as an apprentice to a practitioner in Inverness, and in 1813 went to London and entered as a student in St. George's Hospital under Sir Everard Home and Sir Benjamin Brodie; and he also had the great advantage at Windmill Street Medical School of listening to such teachers as Wilson, Charles Bell, and J. Shaw. The period of student life was then very short, for after spending a year at St. George's he passed the College of Surgeons in 1814, which qualified him to practise at the age of 18 years and five months. Guthrie, who became a great surgeon both in military and civil life, passed the College at the age of 16. Truly these men only began to learn their profession *after* they were qualified. They were simply placed on the rails, they did the rest by their own earnest exertions. Two years later, Ranald Martin, having obtained an appointment as Assistant-Surgeon in the East India Company's service, sailed for Calcutta. Immediately on his arrival he was ordered for duty at the Presidency General Hospital for Europeans. The experience he gained in this hospital laid the foundation of his reputation as an earnest worker and close observer of the diseases of tropical climates, a field fresh and unknown to the European physician.

Having been appointed Assistant-Garrison-Surgeon, he was soon after ordered to accompany a force sent to subdue a revolt in Cuttack, in the district of Orissa. Returning to Calcutta, he was ordered to join the Ramghur Battalion for service in the Gondwana campaign. This was at that time a most unhealthy part of India. Here he suffered from a severe attack of malarial fever; but though unable to walk, he had himself carried about in order to make sanitary inspection, and shortly after he forwarded a report to the commanding officer, advising a move of the troops to a higher and healthier situation. This advice was politely declined, as many others of a similar kind were declined in those days, which resulted, as they usually did, in a higher rate of mortality among the troops. Thus, very early in his military career, did Ranald Martin show that he regarded the prevention of disease as one of the greatest, if not the very greatest, of the duties devolving upon a medical officer in charge of troops.

After only ten years' service in India we find this energetic medical officer, although at the time much broken in health, occupying in the great city of Calcutta a position which augured well for his future success.

In 1830 he was made Presidency Surgeon, and soon after was appointed on the staff of the Native Hospital of Calcutta. It was in this institution that he made his great reputation, not only in medical but in surgical cases: here he devised and first performed the operation for the radical cure of hydrocele by injection of tincture of iodine. In short, after his retirement from army service he became a great general practitioner in Calcutta, and attained the largest practice in the city.

But, on looking at his whole career in India and during his residence in London, it is manifest that the two subjects which were nearest to his heart were his devotion to the interests of the service which he had at first entered, and to the great questions of sanitary science which are so important in preserving the health of armies and communities. When we reflect that when Ranald Martin went to India first the death-rate among Europeans in that country was over 60 per 1000 per annum, and that now, chiefly owing to the work of sanitary reformers, of which Martin was one of the pioneers, the mortality has been reduced to about 18 per 1000, blind indeed must be the chronicler of the latter period of the history of India who does not recognise the blessing which has accrued to native and European alike from the improved health of the army and civil population. The change, too, has been brought about in the face of obstinate prejudices as difficult to overcome in the days that are past among educated European officials, as they are at present among the poor and ignorant natives of Bombay and Calcutta, resisting our attempts to remove from their midst a preventible disease which is decimating their families.

As the climate had begun to tell on his constitution, in 1840 Ranald Martin retired from practice in Calcutta and returned to England, after a service of twenty-five years. His reputation followed him to London, where he took a house in Grosvenor Street, with the intention to practise. He met with the greatest encouragement from Sir Benjamin Brodie, from Guthrie, the President of the Royal College of Surgeons, and others. His success was assured from the first, and he soon attained to a large practice, which he carried on until his sudden death in 1874. In 1859 he was appointed Physician to the India Board in succession to Dr. Scott. This brought him into a more prominent official position, and increased his practice.

About this time also the project of establishing an Army Medical School was taken up by Mr. Sydney Herbert, then Secretary of State for War, and Martin was invited by that minister to become a member of the Senate of the School, which was opened at Fort Pitt, Chatham, in the presence of the War Minister, by the late Sir Thomas Longmore, C.B. In recognition of his great services, Martin was made the recipient of several honours. He received the honour of knighthood, and the Companionship of the Order of the Bath.

"As a practitioner," says Sir John Kaye, "he was one of the most popular that ever entered a sick-chamber. His presence was like a gleam of sunshine, and he seldom quitted a patient without leaving him happier than before." He retired from the active work of his profession in November, 1874, and a few days after he was seized with bronchitis, and passed away after a short illness.

